# Dynamics of chromatin accessibility governing Gd-IgA1 synthesis in B cells associated with IgA nephropathy

**DOI:** 10.1038/s12276-025-01505-1

**Published:** 2025-07-23

**Authors:** Yangang Gan, Jiajia Li, Wenchao Li, Qianqian Han, Rui Zhang, Hao Yu, Weicong Zeng, Fengchu Qing, Manli Luo, Hao Li, Qiongqiong Yang

**Affiliations:** 1 Division of Nephrology, Sun Yat-sen Memorial Hospital, Sun Yat-sen University, Guangzhou, China; 2 Guangdong Provincial Key Laboratory of Malignant Tumor Epigenetics and Gene Regulation, Guangdong–Hong Kong Joint Laboratory for RNA Medicine, Sun Yat-sen Memorial Hospital, Sun Yat-sen University, Guangzhou, China; 3 Medical Research Center, Sun Yat-sen Memorial Hospital, Sun Yat-sen University, Guangzhou, China

**Keywords:** Bioinformatics, IgA nephropathy

## Abstract

IgA nephropathy (IgAN) is the most common primary glomerulonephritis worldwide. B cells are believed to play a central role in the pathogenesis of IgAN by producing galactose-deficient IgA1 (Gd-IgA1), partly due to aberrant gene expression in B cells. However, the mechanisms underlying the abnormal gene expression in B cells derived from patients with IgAN remain elusive. Here we unveil a broad spectrum of variations in chromatin accessibility in B cells of patients with IgAN through an assay for transposase-accessible chromatin using sequencing (ATAC-seq) by evaluating active DNA regulatory components. A total of 629 genes showed transcriptional alterations associated with differentially chromatin accessibility. The degree of chromatin accessibility was associated with gene expression in peripheral blood B cells of patients with IgAN. Gene Ontology analysis of genes associated with differentially expressed genes and differentially accessible regions revealed enrichment in pathways related to the regulation of transcription. Furthermore, KLF4 was also identified as a key transcription factor promoting the production of IgA1 and Gd-IgA1. In vitro, knockdown of KLF4 suppressed the production of Gd-IgA1 in IgA-secreting cell lines. Through RNA sequencing, this study further showed that KLF4 could regulate the expression of genes related to the intestinal immune network for IgA production. Chromatin immunoprecipitation sequencing combined with RNA sequencing revealed that KLF4 could bind to the IL-6 promoter and regulate its expression. Mechanistically, a luciferase reporter assay verified that KLF4 directly bound to the *cis*-regulatory element of IL-6 and promoted its expression. The knockdown of KLF4 was shown to alleviate renal lesions and mesangial hypercellularity in IgAN mice. Collectively, the findings from this study elucidated a chromatin-mediated mechanism underlying the differential responses of B cells in IgAN and identified KLF4 as a potential therapeutic target of IgAN.

## KLF4 drives B cell changes in IgA nephropathy

IgA nephropathy (IgAN) is a common kidney disease in which immune complexes build up in the kidneys, often leading to kidney failure. Researchers explored how B cells contribute to IgAN by studying changes in gene expression and chromatin accessibility. They focused on a protein called KLF4, which influences gene activity. The study involved analyzing blood samples from 20 patients with IgAN and 20 healthy individuals. Researchers used advanced techniques such as RNA sequencing to examine gene expression and chromatin immunoprecipitation sequencing to study protein–DNA interactions. They found that KLF4 plays a important role in producing a harmful form of IgA1, galactose-deficient immunoglobulin A1, involved in IgAN. The results suggest that targeting KLF4 could be a new way to treat IgAN. Future research may explore how KLF4 affects other genes and pathways, potentially leading to better therapies for IgAN.

This summary was initially drafted using artificial intelligence, then revised and fact-checked by the author.

## Introduction

IgA nephropathy (IgAN) is the most prevalent primary glomerulonephritis in the world^1^ and is characterized by the deposition of polymeric galactose-deficient IgA1 (Gd-IgA1) immune complexes in glomeruli^2^. Nearly all patients with IgAN might progress to renal failure during their lifetime^3^. Currently, supportive therapies are the basic treatments for patients with IgAN, with a lack of established disease-specific interventions. Although the pathogenesis of this disease remains to be further elucidated, mucosal immunity plays an important role in IgAN^4^.

B cells can mature into plasma B cells under specific pathogen stimulation in the mucosal immune system, leading to the secretion of antibodies, including IgA1, IgA2 or large polymeric IgA1. These B cells secreting IgA1 antibodies enter the circulation and mucosa, and migrate to the bone marrow and kidneys^5^. Freshly derived B cells from the peripheral blood of patients with IgAN, as well as EBV-immortalized B cells, derived from patients with IgAN immortalized using Epstein–Barr virus, can produce Gd-IgA1^6,7^. B cells play an essential role in the development of IgAN, partly due to abnormal transcriptional expression resulting in high levels of Gd-IgA1^8,9^. Our previous study, along with another using single-cell sequencing, has identified elevated differentially expressed genes (DEGs) in the B cells of patients with IgAN, enriched in viral infection and B cell activation-related pathways^10,11^. Yet, the fundamental molecular mechanisms and key factors governing the transcription and fate determination of B cells secreting Gd-IgA1 remain elusive.

Increasing evidence indicates that epigenetic regulation critically influences cell fate determination by controlling the temporal and cell type-specific expression of genes^12^. Chromatin accessibility, reflecting the degree of chromatin compaction, is an essential feature of chromatin states and is typically acknowledged as a trait of *cis*-regulatory elements, such as transcription factors (TFs), enhancers, insulators, promoters and so on^13^. Chromatin-binding proteins could regulate the accessibility of local DNA by competing against nucleosomes, thereby impacting chromatin states and gene expression, while TFs could be recruited through DNA-specific interactions^14^. By leveraging the sequence characteristics of accessible chromatin regions, it becomes possible to predict TF binding and map dynamic TF regulatory networks^14^. Indeed, accumulating evidence suggests that epigenetic modifications play a key role in IgAN^15^. Hence, by characterizing chromatin alterations and analyzing TF regulatory networks in IgAN, we can gain insight into the epigenetic mechanisms of B cells in IgAN and identify key regulators in the secretion of Gd-IgA1.

In this study, alterations in chromatin accessibility and gene expression were described, and TF regulatory networks in B cells from patients with IgAN were constructed and distinguished, providing a novel standpoint for gaining insight into the pathogenesis of IgAN. KLF4 was also identified as a major TF determining the gene expression program involved in Gd-IgA1 production, demonstrating that KLF4 could promote the synthesis of Gd-IgA1. Our study offers a new perspective for understanding the occurrence and development of IgAN and suggests that KLF4 may serve as a potential therapeutic target.

## Materials and methods

### Study approval

The study was meticulously designed and executed in strict adherence to Chinese law and the principles outlined in the Declaration of Helsinki. The protocol governing the utilization of patient samples in this investigation received approval from the Biomedical Research Ethics Committee of Sun Yat-sen Memorial Hospital (SYSKY-2024-416-02), and explicit informed consent was obtained from all participating individuals. Ethical considerations pertaining to mouse care and experimental procedures were duly addressed, and approved by the Institutional Animal Care and Use Committee of Sun Yat-sen University. All animal care practices and experimental protocols were carried out in accordance with the guidelines determined by the Ethics Committee, with an essential emphasis on minimizing any possible suffering sustained by the animals included in the study.

### Study participants

All participants who were diagnosed with IgAN and involved in this research satisfied the diagnostic criteria determined by the Kidney Disease: Improving Global Outcomes (KDIGO) guidelines^16^. All participants with IgAN were diagnosed via kidney biopsy, and secondary causes of IgA deposition were systematically excluded in accordance with the KDIGO clinical practice guidelines^16^. Healthy controls (HCs) who were meticulously paired with IgAN patients with regard to age and sex were recruited from medical personnel at the same hospital. Thorough information concerning the clinical features and pathological assessments of the enlisted patients are outlined in Table 1.

**Table 1:** Clinical and demographic characteristics of individuals afflicted with IgAN and control participants.

Characteristic	HC (*n* = 20)	IgAN (*n* = 20)	*P* value
Age (years)	31.60 ± 7.30	35.75 ± 14.33	0.256
Male/female	13/7	11/9	0.747
BMI (kg/m^2^)	22.92 ± 2.27	23.26 ± 3.18	0.700
Hypertension	0/20	6/20	0.027
SBP (mmHg)	115.25 ± 10.33	125.70 ± 16.76	0.023
DBP (mmHg)	70.90 ± 8.40	83.25 ± 12.65	0.001
MAP (mmHg)	85.68 ± 6.83	97.4 ± 13.15	0.001
Proteinuria (g/24 h)	n.a.	0.68 (0.26, 1.10)	
Hematuria (RBC/μl)	0.00 (0.00, 0.00)	58 (18.5, 205.5)	<0.001
Hemoglobin (g/l)	135.45 ± 11.45	130.55 ± 20.13	0.350
Serum albumin (g/l)	46.09 ± 2.73	36.80 ± 5.49	<0.001
Uric acid (μmol/l)	396.10 ± 111.98	446.95 ± 134.87	0.202
Serum creatinine (μmol/l)	80.35 ± 13.39	95.45 ± 28.29	0.037
BUN (mmol/l)	4.35 ± 1.12	5.67 ± 2.06	0.016
eGFR (ml/min per 1.73 m^2^)	101.00 ± 17.80	84.14 ± 23.33	0.014
Serum Gd-IgA1 (ng/ml)	4,723.27 ± 1775.46	7,989.4 ± 3392.52	<0.001
Serum IgA1 (g/l)	2.33 ± 0.82	3.12 ± 1.10	0.014
Oxford MEST-C, *n* (%)			
M1	n.a.	19 (95)	
E1	n.a.	3 (15)	
S1	n.a.	13 (65)	
T1/2	n.a.	6 (30)	
C1/2	n.a.	10 (50)	

Results are presented as the mean ± s.d., median (interquartile range) or number (percent). *n*.*a*., not analyzed; *BMI*, body mass index; *SBP*, systolic blood pressure; DBP, diastolic blood pressure; *MAP*, mean arterial pressure; *BUN*, blood urea nitrogen; *eGFR*, estimated glomerular filtration rate; *MEST-C*, M mesangial hypercellularity, *E* endocapillary hypercellularity, *S* segmental glomerulosclerosis, *T* interstitial fibrosis/tubular atrophy, *C* crescent formation.

### Cell isolation

To separate peripheral blood mononuclear cells and B cells, peripheral venous blood was obtained from patients with IgAN and HCs, utilizing heparin as the anticoagulant. Peripheral blood mononuclear cells were obtained using Ficoll Paque Plus (Cytiva). CD19^+^ B cells were isolated using Miltenyi beads (Miltenyi). The purity of CD19^+^ B cells per sample was generally >90% (Supplementary Figs. 2 and 5). For RNA sequencing (RNA-seq) and assay for transposase-accessible chromatin using sequencing (ATAC-seq) analyses, B cell samples from three IgAN and three healthy donors were randomly selected.

### RNA-seq and data analysis

The total RNA from samples was obtained using TRIzol reagent (Invitrogen). Total mRNA was used for generating RNA-seq libraries, which were subsequently sequenced on the NovaSeq platform (Illumina). Gene expression patterns were determined on the basis of read counts. TPM values (fragments per kilobase of exon model per million mapped reads) were used to calculate expression levels and transcript abundance. DEGs were identified through DESeq2 analysis.

### ATAC-seq and data analysis

The quality of cellular preparations was ensured by determining cell viability to be greater than 90% through trypan blue staining. For further analysis, 100,000 cells were lysed in a buffer consisting of 10 mM Tris–HCl (pH 7.4), 10 mM NaCl, 3 mM MgCl_2_ and 0.1% IGEPAL CA-630^17^. The subsequent transposition reaction mixture, incorporating Tn5 transposase, was promptly added to the resuspended nuclear pellet and incubated at 37 °C for 30 min. The transposed DNA fragments were purified using a polymerase chain reaction (PCR) purification kit and eluted in 10 μl of TE buffer. PCR amplification ensued to amplify transposed DNA fragments, involving adapter ligation and quantitative PCR (qPCR) before amplification. Library quality was evaluated through gel electrophoresis, and the libraries, equipped with barcodes, were pooled at equimolar ratios before being sequenced on the NovaSeq platform (Illumina). Subsequently, the peaks were called using MACS2^18^. Differential peak analysis of ATAC-seq data between groups, as well as motif analysis, was performed using HOMER^19^. Visualization of ATAC signals was achieved through a heat map, which was generated using deepTools (v3.4.3).

### In vitro studies utilizing human IgA1-secreting cell lines

B cells derived from patients with IgAN were immortalized using Epstein–Barr virus, subcloned to produce IgA1-secreting cell lines and cultured following established protocols^6^. EBV-immortalized B cells expressed surface CD38 and CD19 molecules after 4 weeks incubation with culture supernatant of B95-8 cells (Supplementary Fig. 1). The cells were cultured in a humidified incubator at 37 °C with 5% CO_2_ in RPMI-1640 medium supplemented with 20% fetal calf serum, streptomycin and penicillin. To downregulate the expression of KLF4 in B cells, B cells were transfected with a specific small interfering RNA (siRNA) for KLF4 (siKLF4) or negative control (siNC) via electrotransfection. Adenoviruses harboring coding sequences for the human KLF4 gene were produced (Hanbio Biotechnology). B cells were exposed to adenovirus particles carrying KLF4 at a multiplicity of infection ranging from 1 × 10^3^ to 1 × 10^4^. Recombinant IL-6, an anti-IL-6 antibody and a control antibody (goat IgG) were procured from R&D Systems. The cells were subjected to 72 h of culture in the presence of 50 ng/ml recombinant IL-6 and 100 ng/ml anti-IL-6 antibody.

### Assays for Gd-IgA1, IgA1 and IL-6 produced by human IgA1-secreting cells

Gd-IgA1 levels were measured using Gd-IgA1 enzyme-linked immunosorbent assay (ELISA) kits (IBL). IgA1 levels were evaluated using a human IgA1 ELISA kit (Cloud Clone), while IL-6 levels were assessed using a human IL-6 ELISA kit (Abclone).

### ChIP-seq and data analysis

Chromatin immunoprecipitation (ChIP) was performed as previously described^20^. Initially, B cells were cross-linked with 1% formaldehyde in phosphate-buffered saline for approximately 15 min at 37 °C. The crosslinking reaction was then halted using 0.125 M glycine. Subsequently, lysis of the cells was carried out using lysis buffer and Bioruptor Pico (Diagenode) was used to generate chromatin fragments within the size range of 100–800 bp. A 10% fraction of the sonicated chromatin sample was reserved for the input control, while the rest of the chromatin was subjected to overnight incubation with an antibody at 4 °C. After elution and reverse crosslinking, the DNA fragments were subjected to library preparation and sequenced on the NovaSeq platform (Illumina). ChIP sequencing (ChIP-seq) data were analyzed as previously outlined^20^. ChIP-seq peak calling and BED file generation were performed using MACS2. Differentially accessible regions (DARs) were analyzed using HOMER based on normalized trimmed counts. In addition, HOMER was used for the annotation of peaks.

### ChIP–qPCR

The ChIP experiment was conducted following the previously outlined procedure. ChIP assays were performed using the anti-KLF4 antibody (R&D Systems). Immunoprecipitation was conducted with the specified antibody, and 10% of the supernatants were retained as input. Rabbit normal serum IgG was used as a control. For ChIP–qPCR, DNA from the immunoprecipitated and input groups was evaluated using real-time PCR with specific primers.

### Quantitative real-time PCR

Total RNA was extracted from B cells using the TRIzol reagent (Invitrogen). Subsequently, the isolated RNA was utilized for reverse transcription, generating cDNA, with the PrimeScript RT kit (Takara). Real-time PCR was conducted on the LightCycler 384 System (Roche) using SYBR Green Master Mix (Takara). Relative expression levels were calculated using the 2^−ΔΔCT^ method, with GAPDH serving as the normalization control. All primers indicated in Supplementary Table 1 were procured from IGE Biotechnology.

### Luciferase reporter assay

HEK-293T cells were co-transfected with a luciferase reporter plasmid and either KLF4 or a negative control using Lipofectamine 3000 (Invitrogen). After 48 h, the luciferase activity was measured using a Dual-Glo Luciferase Reporter Assay System (Promega) as per the manufacturer’s instructions. The firefly luciferase activity in each sample was then normalized to the renilla luciferase activity.

### Western blotting

Protein lysates from IgA1-secreting cells were analyzed using 12% SDS–PAGE gels. The separated proteins were then transferred into polyvinylidene fluoride membranes and probed with antibodies specific for KLF4 and GAPDH. Chemiluminescence detection was carried out using enhanced chemiluminescence (Affinity) and a luminescence detector (Thermo). Densitometric analysis was performed to evaluate the results.

### Animal models

Balb/c mice, aged 6 or 8 weeks old (weighing 25 ± 5 g), were procured from the Guangdong Provincial Laboratory Animal Center (Guangzhou, China). The IgAN mouse model was induced following previously established procedures^21–23^. Mice were given oral administration of 0.1% bovine serum albumin (Sigma) with acidified water, at a dosage of 0.4 ml per mouse, on alternate days. After 6 weeks, the mice were injected with 0.1 ml of 1% bovine serum albumin buffer solution via the tail vein once daily for 3 consecutive days. From the 9th week onward, the mice received a weekly injection of staphylococcal enterotoxin B (the Academy of Military Medical Sciences, China), at a dose of 0.4 mg/kg for 3 weeks. Acidified water was orally administered to the control group on alternative days until death. The control group was provided with acidified water orally on alternate days until reaching the endpoint. They were administered injections of saline via tail veins, following the same dosage and schedule as the model group. At the 13th week time point, all Balb/c mice were humanely euthanized.

### Gene delivery in animals

A pair of AAV-DJ vectors was used to facilitate B cell engineering in vivo^24^. Subsequently, short hairpin RNA (shRNA) was used under the control of CD19 to enhance safety^25^. After sequencing to ensure the accuracy of the AAV/DJ vector, it was packaged, purified and titrated (GeneChem). The AAV/DJ vector carrying either shKLF4 (AAV-shKLF4) or the control sequence (AAV vector) was injected intravenously with 5 × 10^11^ viral genomes (vg)/100 µl/mouse in phosphate-buffered saline. The injection was administered at the 6th week.

### In vivo quantification of urinary albumin and creatinine concentrations

Spot-urine samples were collected, and the levels of urinary albumin and creatinine were measured using urinary albumin ELISA kits (Elabscience) and creatinine ELISA kits (Elabscience), respectively.

### Quantification of IgA in murine serum

Serum IgA were measured using a mouse IgA ELISA kit (Jonln) following the manufacturer’s protocols.

### Histological analyses of renal tissues

Kidneys were retrieved following perfusion with normal saline. Renal tissues were fixed in 4% paraformaldehyde, embedded in paraffin and sectioned into 3-μm-thick slices. Periodic acid–Schiff staining was performed to assess mesangial cell proliferation and matrix expansion. The stained renal tissue sections were examined and scored by pathologists under an optical microscope. IgA deposition was analyzed using immunofluorescence as previously outlined^26^. We used a 1:50 dilution of fluorescein isothiocyanate (FITC)-labeled goat anti-mouse IgA (Abcam) to identify IgA in renal tissues, and imaging was performed using laser scanning confocal microscopy (Leica).

### Statistical analysis

The statistical analyses and graphs for this research were conducted using GraphPad Prism 10.0 and R 4.2.3. Data were presented as mean ± s.d., mean ± s.e.m. or median values. Chi-square tests were applied for comparisons of categorical variables. For normally distributed quantitative variables, two-tailed Student’s *t*-tests and Pearson correlation coefficients were used. Nonnormally distributed variables were analyzed using the Mann–Whitney *U* test and Spearman correlation coefficients. Multiple group comparisons were assessed through one-way ANOVA. *P* < 0.05 was considered statistically significant.

## Results

### Clinical and demographic characteristics of individuals afflicted with IgAN and control participants

The study population comprised 40 participants, including 20 patients with IgAN and 20 HCs. The demographic and clinical characteristics of these participants are presented in Table 1. The average age of patients with IgAN was 35.75 ± 14.33 years, which was comparable to that of the HCs (31.60 ± 7.30 years) (*P* > 0.05). Notably, IgAN patients exhibited higher levels of systolic blood pressure, diastolic blood pressure, 24-h proteinuria, creatinine, urea nitrogen, Gd-IgA1 and IgA1 compared with HC (*P* < 0.05). The levels of baseline estimated glomerular filtration rate were significantly lower than those in HCs (*P* < 0.05).

### The alterations of chromatin accessibility and gene expression in peripheral blood B cells of IgAN

**Fig. 1:**
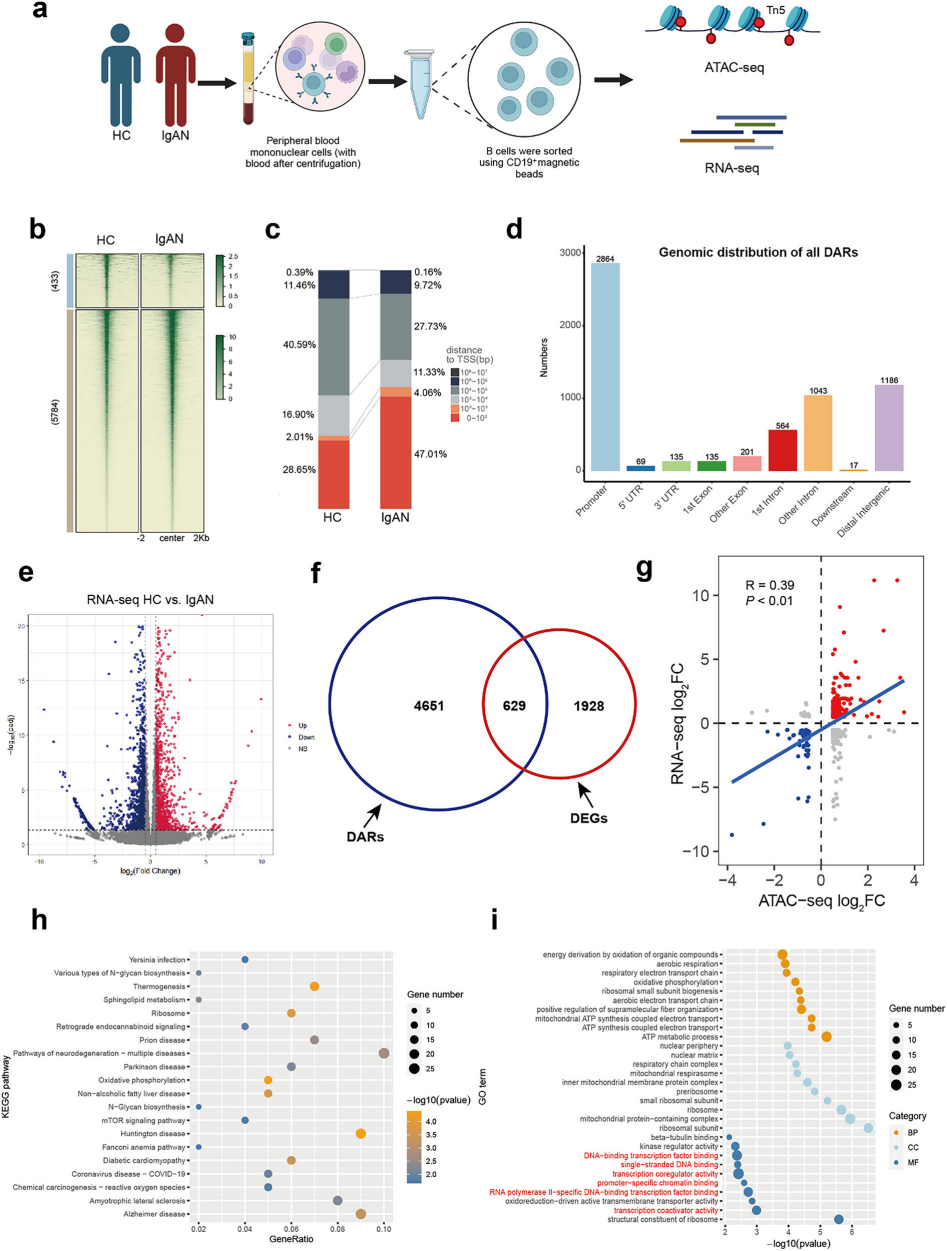
The alterations of chromatin accessibility and gene expression in peripheral blood B cells of patients with IgAN. **a** An overview of the experimental approach used for genome-wide ATAC-seq and RNA-seq analysis. **b** Alterations of chromatin accessibility in B cells of patients with IgAN, with peaks organized in a vertical order based on ATAC-seq signal strength, represented by varying color intensities. **c** Proximity analysis of transcription start sites (TSSs) highlighting the distances between accessible regions in the HC and IgAN groups. **d** A histogram chart displaying the annotation results of DAR peaks. **e** Visualization of transcriptome data through a scatter plot (log_2_ fold change (FC) > 0.5, adjusted *P*-value < 0.05). **f** A Venn diagram depicting the intersection between genes related to DARs and DEGs. **g** Scatter plots of the genes associated with DEGs and DARs. Red indicates genes that exhibit increased accessibility and upregulation in patients with IgAN, and blue indicates genes that are more accessible and upregulated in HCs. **h** KEGG pathway analysis focusing on genes associated with DEGs and DARs. **i** GO pathway analysis focusing on genes associated with DEGs and DARs.

To characterize the chromatin landscape in peripheral blood B cells of IgAN, ATAC-seq analysis was conducted on B cells obtained from three patients with IgAN and three HCs (Fig. 1a). DARs in B cells from HCs and patients with IgAN were distinguished using a clustering approach. Both lost and gained genome accessibility regions were observed in IgAN group (Fig. 1b). The majority of the identified accessible areas in IgAN were enriched in the transcription start site (Fig. 1c), with a significant portion located in promoter regions (Fig. 1d). For a deeper understanding of the biological processes related to these DARs, each DAR peak was assigned to the nearest gene.

To investigate whether differences in chromatin openness corresponded to gene expression in peripheral blood B cells of HCs and patients with IgAN, RNA-seq was conducted on the same samples in the ATAC-seq analysis. Using the defined criteria for DEGs, 1138 upregulated and 1,419 downregulated mRNAs were identified (Fig. 1e). To explore the relationships between gene expression and chromatin accessibility, an analysis that integrated ATAC-seq and RNA-seq data was conducted. A total of 629 genes demonstrated transcriptional alterations associated with differential chromatin accessibility (Fig. 1f). Moreover, the integrated analysis revealed a correlation between alterations in chromatin accessibility and variations in gene expression patterns in peripheral blood B cells (Fig. 1g). Chromatin accessibility is one of the reasons for the abnormal expression of B cell genes in patients with IgAN. Kyoto Encyclopedia of Genes and Genomes (KEGG) analysis of genes associated with DEGs and DARs showed enrichment in various viral and bacterial infection-related pathways (Fig. 1h). Gene Ontology (GO) analysis of genes associated with DEGs and DARs revealed enrichment in pathways related to the regulation of transcription (Fig. 1i).

### Enrichment of KLF4 motif in DARs of peripheral blood B cells of IgAN

**Fig. 2:**
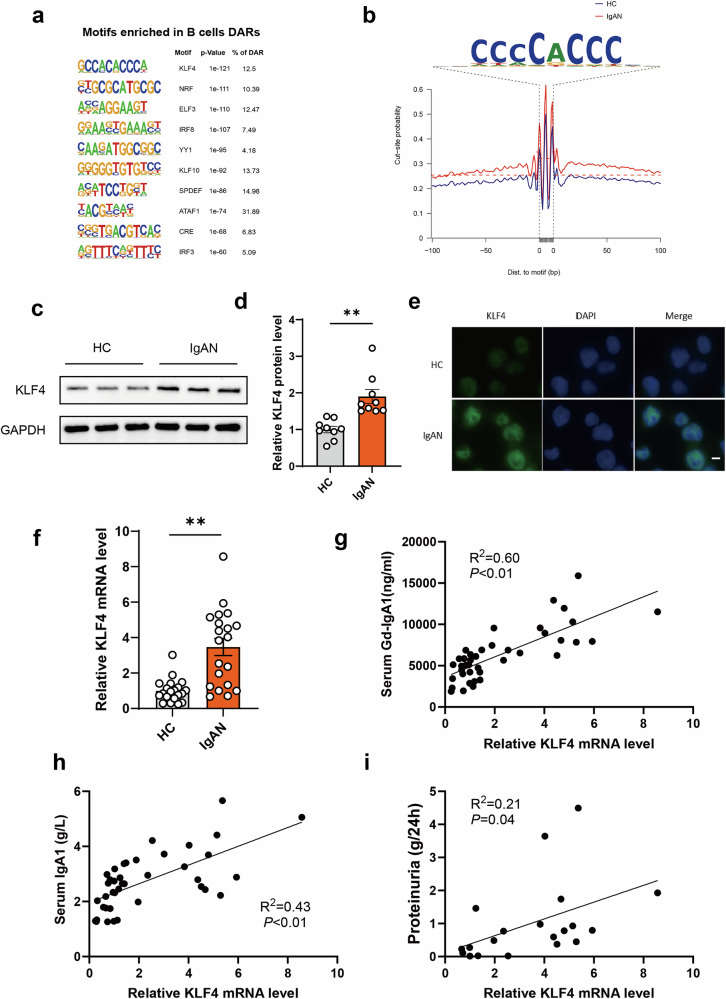
KLF4 as a putative transcriptional regulator in peripheral blood B cells of patients with IgAN. **a** Enrichment of motifs within DARs indicative of KLF4 involvement. **b** ATAC-seq footprint analysis demonstrating the full site occupancy of KLF4 in peripheral blood B cells of HC and IgAN groups. **c**,**d**, Validation of KLF4 protein expression in peripheral blood B cells from the HC and IgAN groups through western blot analysis. **e**, Immunofluorescence staining of KLF4 comparing the expression levels in peripheral blood B cells between HC and IgAN groups. **f**, Verification of KLF4 mRNA expression in peripheral blood B cells from HC and IgAN groups using qPCR. **g**, **h** Correlation of relative mRNA levels of KLF4 with serum levels of Gd-IgA1 and IgA1. **i** Correlation of relative mRNA levels of KLF4 with the level of proteinuria in patients with IgAN. Bars represent mean ± s.e.m. *P < 0.05, **P < 0.01.

Chromatin accessibility serves as a marker of regulatory regions and is typically required for the binding of TFs to regulate gene expression^27^. To pinpoint enriched TFs implicated in the regulation of gene expression, motif enrichment analysis was conducted to identify TFs that target distinct chromatin states^28^. Subsequently, motifs within the DAR-associated genes were forecasted to pinpoint the TFs responsible for governing the expression of these genes. The top TF-targeting motifs are shown in Fig. 2a. Prominent evidence of TF binding was observed surrounding the consolidated KLF4 binding sites within accessible regions, indicating a long-lived interaction between KLF4 and DNA (Fig. 2b). Given that the mechanism and function of KLF4 in IgAN have not been previously reported, focus was placed on KLF4 for further analysis. The expression levels of KLF4 in both HC and IgAN groups were subsequently examined in a large number. The expression of KLF4 was higher in IgAN groups than in HC groups, as verified by western blot, qPCR and immunofluorescence staining (Fig. 2c–f). The relative mRNA levels of KLF4 in peripheral blood B cells were positively associated with serum levels of Gd-IgA1 and IgA1 (Fig. 2g, h). Furthermore, the relative mRNA levels of KLF4 in peripheral blood B cells were also positively correlated with the degree of proteinuria in patients with IgAN (Fig. 2i). Collectively, these findings suggest that KLF4 in peripheral blood B cells may promote the production of Gd-IgA1 in IgAN.

### KLF4 knockdown or overexpression in B cells on the synthesis of Gd-IgA1 in vitro

**Fig. 3:**
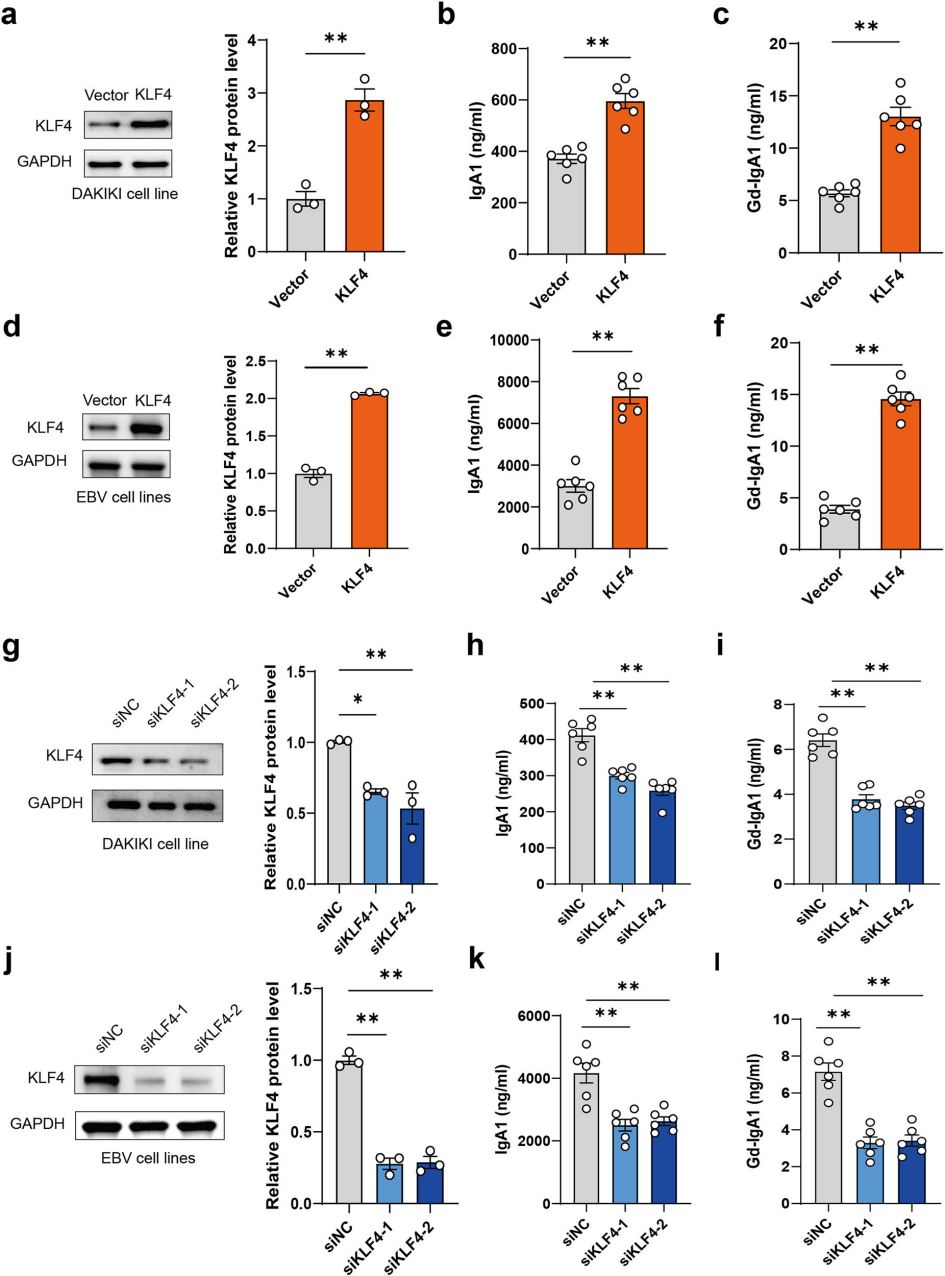
Effects of KLF4 overexpression and knockdown on the synthesis of Gd-IgA1 in vitro. **a** Transfection of the DAKIKI cell line with adenoviruses containing coding sequences for KLF4, followed by western blot analysis to assess target protein expression. **b**, **c** Collection of culture supernatant 3 days after transfection for IgA1 (**b**) and Gd-gA1 (**c**) ELISA analysis (*n* = 6). **d** Transfection of EBV-immortalized B cells with adenoviruses containing coding sequences for KLF4, followed by western blot analysis for target protein expression. **e**, **f** Collection of culture supernatant from EBV-immortalized B cells 3 days after transfection for IgA1 (**e**) and Gd-gA1 (**f**) ELISA analysis (*n* = 6). **g** Electroporation of synthetic siRNA targeting the human KLF4 gene or control siRNA into the DAKIKI cell line. **h**, **i** Collection of culture supernatant from the DAKIKI cell line 3 days after electroporation for IgA1 (**h**) and Gd-gA1 (**i**) ELISA analysis (*n* = 6). **j**, Electroporation of synthetic siRNA targeting the human KLF4 gene or control siRNA into the EBV-immortalized B cell lines. **k**, **l** Collection of culture supernatant from EBV-immortalized B cell lines 3 days after electroporation for IgA1 (**k**) and Gd-gA1 (**j**) ELISA analysis (*n* = 6). Bars represent the mean ± s.e.m. *P < 0.05, **P < 0.01.

Next, to examine the function of KLF4 in B cells on the synthesis of Gd-IgA1 in vitro, adenoviruses carrying coding sequences for KLF4 were transfected into the DAKIKI cell line and EBV-immortalized B cell lines (Fig. 3a, d). The overexpression of KLF4 led to a significant increase in the secretion of IgA1 and Gd-IgA1 in DAKIKI cell line (Fig. 3b, c). The overexpression of KLF4 also promoted the secretion of IgA1 and Gd-IgA1 in EBV-immortalized B cell lines (Fig. 3e, f). To further validate these findings, synthetic siRNA targeting the human KLF4 gene or negative control was electroporated into the DAKIKI cell line and EBV-immortalized B cells (Fig. 3g, j). Knockdown of KLF4 expression significantly diminished the secretion of both IgA1 and Gd-IgA1 in the DAKIKI cell line (Fig. 3h, i). Knockdown of KLF4 expression significantly decreased the secretion of both IgA1 and Gd-IgA1 in EBV-immortalized B cell lines (Fig. 3k, l). KLF4 also significantly increased the percentage of Gd-IgA1 in total IgA1 in vitro (Supplementary Fig. 3). These data provide further evidence supporting the role of KLF4 in regulating the synthesis of Gd-IgA1.

### KLF4 promotes the expression of genes related to the intestinal immune network for IgA production

**Fig. 4:**
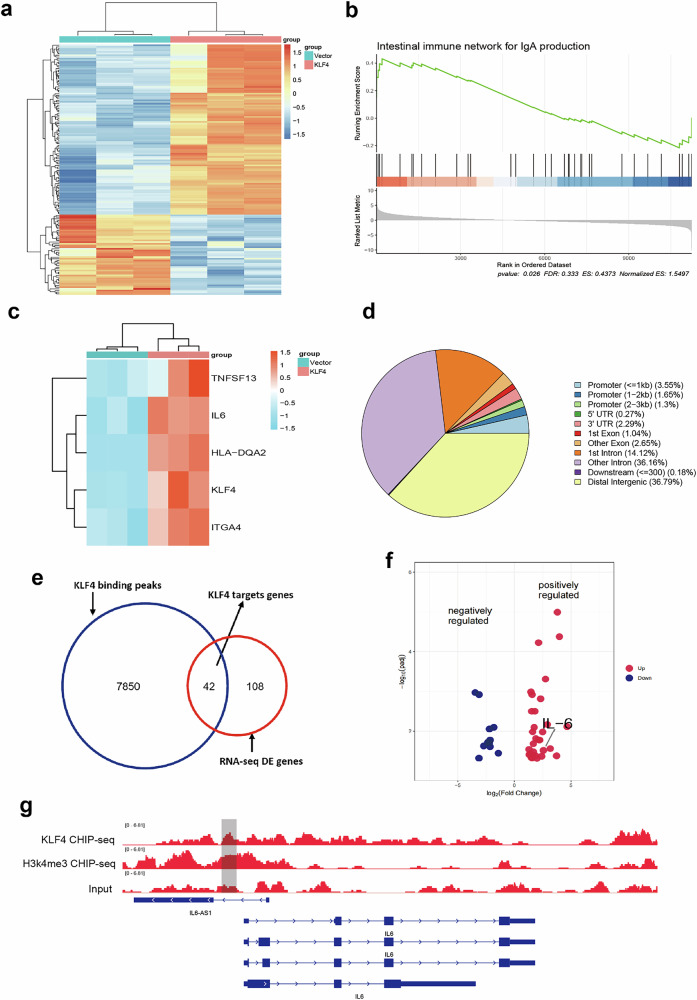
Genome-wide expression profiling identified IL-6 as a KLF4 target. **a** A heat map illustrating DEGs identified through bulk RNA-seq of EBV-immortalized B cells transfected with adenoviruses carrying coding sequences for the KLF4 or vector gene. **b** GSEA highlighting the intestinal immune network for IgA production pathways enriched in KLF4-overexpressing EBV-immortalized B cells. **c** Heat maps of genes involved in the intestinal immune network for the IgA production pathway. **d** Genomic distribution of KLF4 binding peaks across the B cell genome. **e** A Venn diagram showing the overlap between KLF4-regulated genes and KLF4-bound genes. **f** Volcano plots displaying the expression levels of KLF4 target genes in KLF4-overexpressing B cells. Red indicates increased expression, while blue signifies reduced expression, with highlighted representative genes. **g** Representative alignments of ChIP-seq data illustrating KLF4 and H3k4me3 binding to the specified loci in B cells.

The precise regulation of gene expression is vital for the growth of the body and the maintenance of various physiological functions of cells. Disorders in gene expression affect numerous physiological and pathological processes, with transcription being the most critical step in the regulation of gene expression. To further explore the potential therapeutic targets of KLF4, RNA-seq analyses were conducted to detect EBV-immortalized B cells transfected with adenoviruses carrying coding sequences for KLF4 or vector control. Heat maps of RNA-seq showed the DEGs of KLF4 overexpression and vector control (Fig. 4a). Gene set enrichment analysis (GSEA) of the RNA-seq data suggested an enrichment of the intestinal immune network for IgA production in KLF4-overexpressing EBV-immortalized B cells (Fig. 4b). This network included key genes such as HLA-DQA2, IL-6, ITGA4 and TNFSF13 (Fig. 4c). Previous GWAS studies have identified associations between HLA-DQA, TNFSF13, IL-6 and susceptibility to IgAN^29^. Moreover, we found that the NF-κB signaling pathway, B cell receptor signaling pathway, protein–DNA complex, immunoglobulin complex and transcriptional regulation-related pathways were enriched in KLF4-overexpressing EBV-immortalized B cells (Supplementary Fig. 4). Our data suggested that KLF4 could affect the inflammation response, B cell function and transcriptional regulation. Notably, IgAN could induce KLF4, which interacts with gene regulatory regions and promotes the expression of genes involved in IgA production.

### KLF4 promotes B cell secretion of IgA1 and Gd-IgA1 via the KLF4–IL-6 axis

**Fig. 5:**
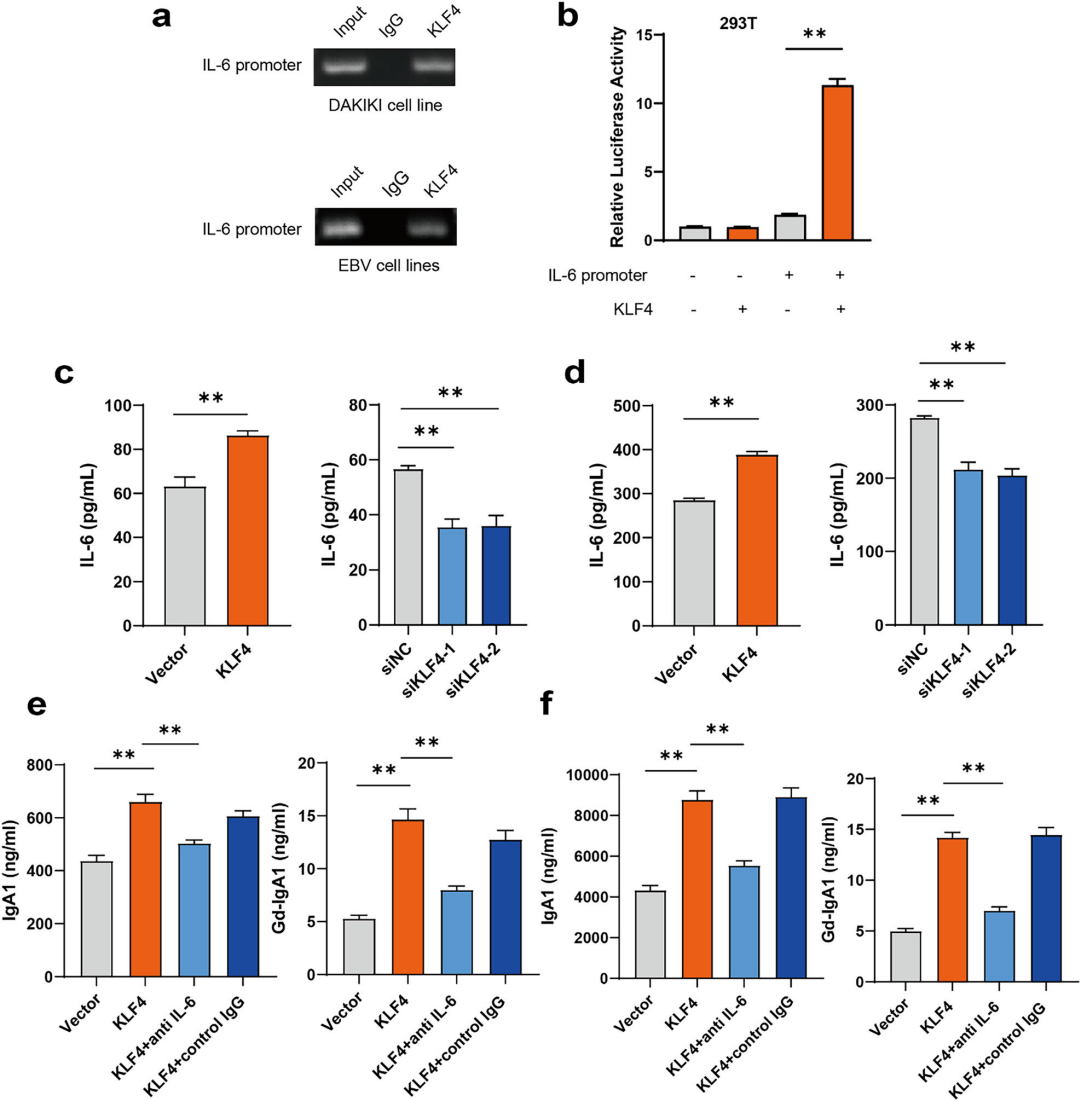
KLF4 promotes B cells secretion of IgA1 and Gd-IgA1 via the KLF4–IL-6 axis. **a** ChIP assays conducted in DAKIKI and EBV cells revealed KLF4 binding sites across the IL-6 promoter region. **b** Evaluation of luciferase activity in IL-6 promoter constructs after transfection of the KLF4 plasmid into 293T cells. **c** Expression of IL-6 in the DAKIKI cell line under conditions of KLF4 overexpression and knockdown of KLF4. **d** Expression of IL-6 in EBV-immortalized B cells under conditions of KLF4 overexpression and knockdown of KLF4. **e** Partial reduction in the enhanced production of IgA and Gd-IgA1 induced by KLF4 through treatment with anti-IL-6 antibody in the DAKIKI cell line. **f** Partial reduction in the enhanced production of IgA and Gd-IgA1 induced by KLF4 through treatment with an anti-IL-6 antibody in EBV-immortalized B cells. Bars represent the mean ± s.e.m. *P < 0.05, **P < 0.01.

Next, ChIP-seq was used to detect the binding sites of KLF4 across the genome. The binding sites of KLF4 were mainly distributed in the distal intergenic regions (Fig. 4d). An intersection analysis with the ChIP-seq and RNA-seq data was performed, which identified 42 direct targets of KLF4 (Fig. 4e). The pathway of intestinal immune network for IgA production plays an important role in the occurrence and development of IgAN. Therefore, we further narrowed the scope to the intestinal immune network for the IgA production pathway and found that KLF4 could directly target IL-6 (Fig. 4f). Notably, KLF4 directly regulates the expression of IL-6 by binding to its promoter region, which is also involved in the intestinal immune network for IgA production (Fig. 4g). In DAKIKI and EBV cells, the ChIP assay revealed the presence of KLF4-binding sites across the IL-6 promoter region (Fig. 5a). After transfection of the KLF plasmid, the luciferase activity of IL-6 promoter was observed to be notably high in 293T cells (Fig. 5b). The expression of IL-6 in the DAKIKI cell line was observed to be upregulated under KLF4 overexpression, while its expression was lower in the condition of KLF4 knockdown (Fig. 5c). The expression of IL-6 in EBV-immortalized B cells was observed to be upregulated under KLF4 overexpression, while its expression was lower in the condition of KLF4 knockdown (Fig. 5d).

**Fig. 6:**
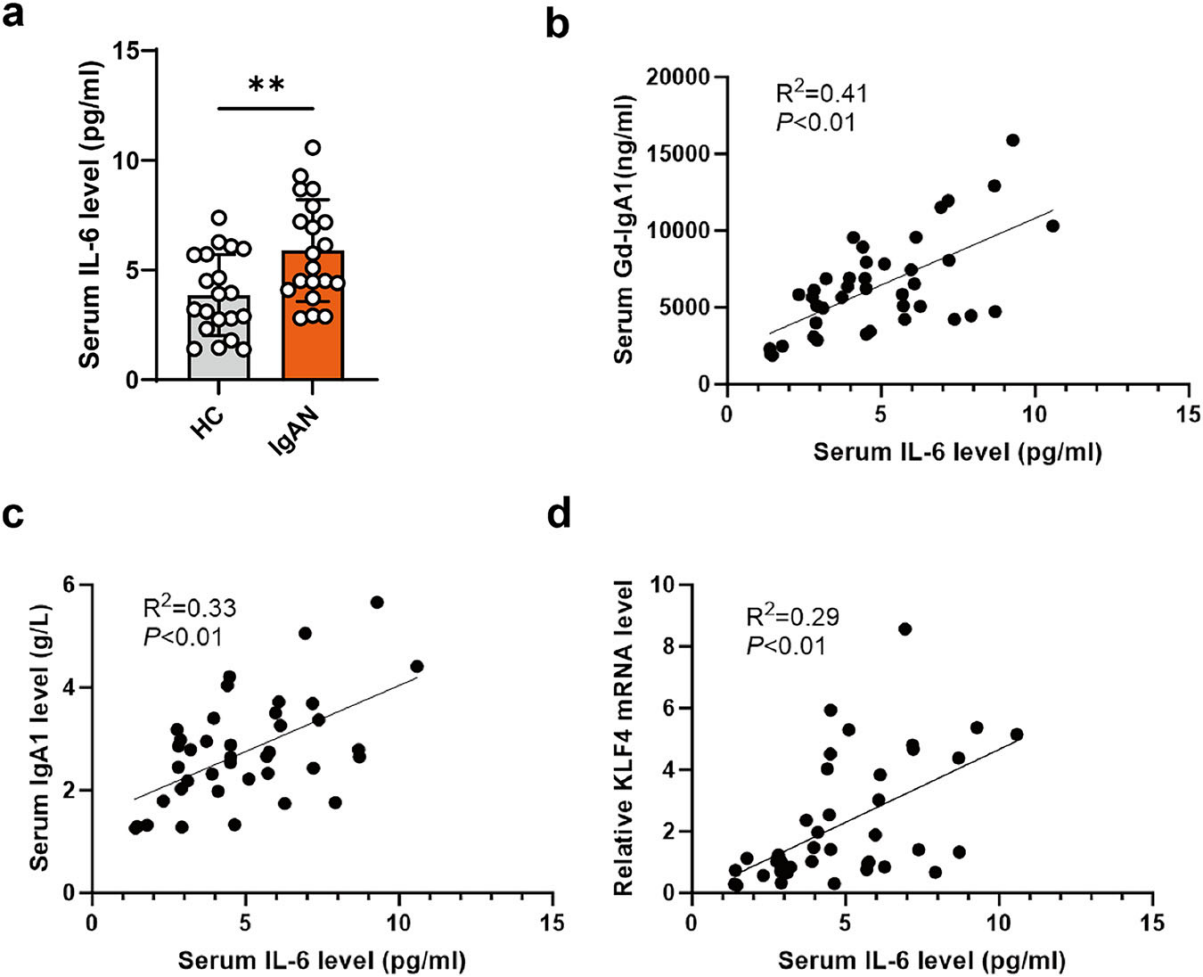
Serum IL-6 levels were high and associated with serum Gd-IgA1 in patients with IgAN. **a** Serum IL-6 levels were higher in patients with IgAN than in HCs. **b** Association between serum IL-6 levels and Gd-IgA1. **c** Correlation of serum IL-6 levels with serum IgA1 levels. **d** Association of serum IL-6 levels with KLF4 mRNA levels in B cells. Bars represent mean ± s.e.m. *P < 0.05, **P < 0.01.

The production of IgA1 and Gd-IgA1 induced by KLF4 was partially reduced by the anti-IL-6 antibody in the DAKIKI cell line (Fig. 5e) and EBV-immortalized B cells (Fig. 5f). These results suggest that KLF4 promoted the generation of Gd-IgA1 probably through the KLF4–IL-6 axis. Serum levels of IL-6 were found to be elevated in the IgAN groups (Fig. 6a). Previous studies have identified IL-6 as the most significant cytokine involved in the synthesis of Gd-IgA1 in IgAN^30^. In this study, we also found that serum IL-6 levels were also positively correlated with serum Gd-IgA1 and IgA1 (Fig. 6b, c). Moreover, relative serum IL-6 levels were positively associated with KLF4 mRNA in B cells (Fig. 6d).

### KLF4 knockdown in B cells mitigated renal injury in IgAN mice via decreased production of IgA

**Fig. 7:**
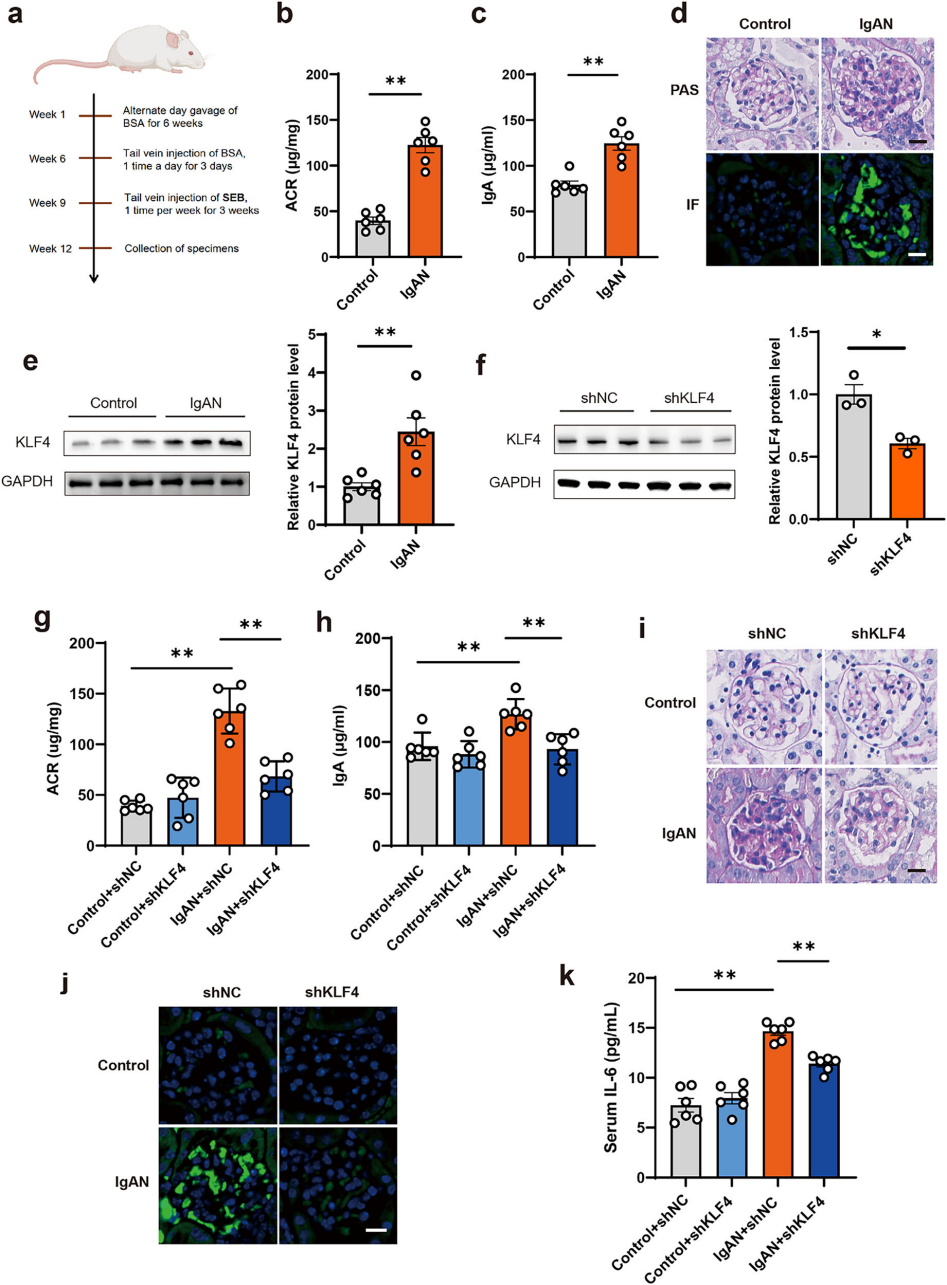
Targeted knockdown of KLF4 expression in IgAN mice B cells attenuates IgA secretion and IgA deposition. **a** Flow chart of IgAN mouse modeling. **b** Spot-urine samples were collected at the end of 12 weeks for ACR analysis. **c** Serum IgA levels in the control and IgAN groups were measured. **d** Mesangial proliferation and mesangial deposits of IgA in the glomerular area of IgAN mice. **e** Western blot analysis showing the upregulation of KLF4 protein in B cells from IgAN mice. **f** Lower expression of KLF4 was observed in the shKLF4 group compared with the shNC group in vivo. **g** Reduced proteinuria was observed in IgAN mice with KLF4 knockdown. **h** Serum IgA levels were decreased in IgAN mice with KLF4 knockdown. **i** Periodic acid–Schiff (PAS) staining images of glomeruli. Scale bars, 20 μm. **j** Confocal images of immunofluorescence. IgA-FITC (green) and DAPI were used to stain the nuclei (blue). Scale bars, 20 μm. **k** Serum levels of IL-6 were decreased in IgAN mice with KLF4 knockdown. Bars represent the mean ± s.e.m. *P < 0.05, **P < 0.01.

In this section, we aim to establish a mouse model of IgAN and investigate the role and mechanism of KLF4 in the pathogenesis of IgAN (Fig. 7a). Compared with the control group, the urea nitrogen and serum creatinine levels in the IgAN group were slightly higher but did not reach statistical significance (Supplementary Fig. 6). The urinary albumin/creatinine ratio (ACR), serum IgA levels, mesangial proliferation and IgA intensity were increased in the IgAN group, which confirmed the successful establishment of the IgAN mouse models (Fig. 7b–d). The protein levels of inflammatory markers were also increased in kidney tissue from IgAN mice (Supplementary Fig. 7). The protein level of KLF4 was significantly upregulated in B cells of IgAN mouse model (Fig. 7e). To investigate the effect of KLF4 in B cells from a murine model of IgAN, AAV/DJ of shRNA injection was used to suppress the expression of KLF4 in B cells of IgAN mice (Fig. 7f). The knockdown of KLF4 in B cells of IgAN mice resulted in a significant reduction in proteinuria and serum IgA levels (Fig. 7g, h). Mesangial proliferation and mesangial matrix expansion were reduced upon knockdown of KLF4 in B cells of the IgAN group (Fig. 7i). Mesangial deposits of IgA were also reduced upon knockdown of KLF4 in B cells of the IgAN group (Fig. 7j). The knockdown of KLF4 in B cells of IgAN mice also resulted in a significant reduction in serum IL-6 levels (Fig. 7k). These findings indicate that the suppression of KLF4 in B cells of mice can effectively mitigate the kidney injury caused by IgAN.

## Discussion

B cells play a crucial role in the pathogenesis of IgAN, partly due to abnormal transcriptional expression that leads to a high level of Gd-IgA1^8,9^. Elucidating the mechanisms about the synthesis of nephritogenic IgA in IgAN is of significance for the treatment of IgAN. Despite all efforts, however, the specific mechanism of the increase of Gd-IgA1 in patients with IgAN remains unclear. At present, the alterations of genome-wide chromatin accessibility in B cells of patients with IgAN are not yet fully understood. Through mapping active DNA regulation elements by ATAC-seq, extensive chromatin remodeling in IgAN was demonstrated, which was also associated with the expression of pathogenic genes. This finding indicates that chromatin accessibility alteration may be a promising therapeutic approach for managing IgAN. By analyzing TFs, we identified KLF4 as a key regulator in Gd-IgA1 production of B cells in patients with IgAN.

Alterations of chromatin accessibility govern the expression of a wide array of genes, thereby exerting influence on multiple signaling pathways^14,31^. Chromatin remodeling can achieve systemic effects by simultaneously modulating the expression of multiple pathogenic genes and signaling pathways, instead of regulating only one gene or signaling pathway. The degree of chromatin accessibility is strongly associated with diverse *cis*-regulatory elements^32–34^. Mounting evidence suggests that epigenetic mechanisms, such as DNA methylation, histone modifications and diverse noncoding RNAs, play crucial roles in the development of IgAN^35–37^. In this study, we found that the degree of chromatin accessibility was associated with gene expression. GO analysis of genes associated with DEGs and DARs revealed enrichment in pathways related to the regulation of transcription. The results showed that there was abnormal transcriptional regulation in peripheral blood B cells of patients with IgAN. KEGG analysis of genes associated with DEGs and DARs showed enrichment in various viral and bacterial infection-related pathways. This further suggested that the B cells of patients with IgAN may have viral and bacterial infections. This study highlights the alteration of chromatin accessibility in B cells of patients with IgAN, enhancing our understanding of the epigenetic processes involved in IgAN.

Chromatin accessibility also serves as a crucial marker for regulatory regions and is typically required for the binding of TFs to regulate gene expression^27^. Through motif enrichment and footprint analysis, KLF4 was identified as a key gene in peripheral blood B cells of patients with IgAN. The Kruppel-like factor family comprises zinc finger protein TFs that participate in the regulation of cell proliferation, embryonic development, cell differentiation and cell cycle arrest^38^. KLF4 plays different biological roles after binding to different target genes, exhibiting dual functions of transcriptional activation or inhibition^38^. In renal tissue, KLF4 is expressed in podocytes, which plays a protective role^39^. KLF4 could also promote renal fibrosis in mice after ischemia–reperfusion kidney injury^40^. KLF4 in macrophage ameliorates CKD by mitigating TNF-dependent injury and fibrosis^41^. A spatial transcriptomic study of IgAN patients with mesangial hyperplasia showed decreased KLF4 expression and increased markers of podocyte injury in renal tissue of patients with IgAN^42^. Therefore, KLF4 is a multifunctional TF that exerts different roles in different tissues and cells. In immune cells, KLF4 can increase the survival rate of natural killer cells and maintain the homeostasis of the number of dendritic cells in the spleen^43^. KLF4 influences tumor cells by regulating M2-type macrophage polarization in hepatocellular carcinoma^44^. With regard to B cells, early studies have shown that KLF4 directly promotes cyclin D2 to regulate B cell number and cell proliferation^45^. KLF4 could also promote the generation of mature plasma cells^46^. In this study, the expression of KLF4 was also demonstrated to be high in peripheral blood B cells of patients with IgAN. Consistent with our previous studies, single-cell transcriptome sequencing revealed that KLF4 was highly expressed in the peripheral B cells of patients with IgAN^10^. In addition to KLF4, IRF8 was also found to be highly enriched in IgAN-DARs, and it has previously been shown to be involved in IgAN^47^. These studies will contribute to uncovering the role of KLF4 in IgAN and Gd-IgA1 synthesis, potentially leading to new strategies and targets for the treatment and prevention of this disease.

KLF4 can bind to transcription regulatory elements, including promoters and enhancers to regulate gene expression^48^. With advancements in technology, ChIP-seq has become a simple tool for studying TF binding. To conduct KLF4 gain-of-function experiments and obtain an ample number of cells for ChIP-seq analyses, we used B cells that were transduced with a KLF4-encoding vector. The overexpression of KLF4 increased the production of Gd-IgA1 and IgA1 in B cell lines. Indeed, silencing KLF4 expression with siRNA can reduce the production of Gd-IgA1 and IgA1. These analyses demonstrated that KLF4 could enhance the production of IgA1 and Gd-IgA1. To further identify the target genes regulated by KLF4, we performed RNA-seq on B cell lines under the overexpression of KLF4. The enrichment pathway identified by GSEA was the intestinal immune network for IgA production in the context of KLF4 overexpression. The combined analysis of ChIP-seq and RNA-seq revealed that KLF4 could regulate the expression of IL-6 by binding to its promoter region. Mechanistically, a luciferase reporter assay confirmed that KLF4 directly binds to the *cis*-regulatory element of IL-6 and promotes its expression. IL-6 is recognized for its role in promoting the proliferation and differentiation of B cells^49–51^ and may also be involved in T follicular helper cell differentiation^52,53^. T follicular helper cells play a critical role in the affinity maturation of B cells, class switch recombination, and generation of memory B cells and plasma cells during germinal center differentiation^52,53^. Findings from this study demonstrated that IL-6 may be a important factor in the excessive production of nephritogenic IgA caused by KLF4. Finally, we further validated the role of KLF4 in vivo. After inhibiting the expression of KLF4 in B cells of IgAN mice, the levels of proteinuria, serum IgA, IL-6 and the IgA deposited in the mesangial region of IgAN mice decreased.

Our findings can improve our understanding of the mechanism of disease-causing gene expression in B cell of patients with IgAN. Many potential regulatory targets of KLF4, such as IL-6 and APRIL, have also been identified as variant loci in GWAS of IgAN. The majority of single-nucleotide polymorphisms discovered through GWASs are located in noncoding regions^54^. Understanding the landscape of transcription regulatory elements in IgAN will enhance the ability to connect variants identified through GWAS to specific genes and discover new target genes. Future research should involve CRISPR interference or base editing techniques, as well as novel approaches such as pooled CRISPR screens and single-cell RNA-seq. These investigations are necessary to explore the mechanism of genes expression in IgAN and ascertain their contribution to disease prevention or progression. The specific mechanism by which KLF4 regulates the production of Gd-IgA1 in B cells remains to be further explored. Previous studies have shown that IL-6 can promote the production of Gd-IgA1 by inhibiting the expression of galactosyltransferase^55,56^. Whether KLF4 can regulate galactosyltransferase to induce the production of Gd-IgA1 requires further research in the future.

In conclusion, an analysis was conducted on chromatin accessibility and gene expression profiles in peripheral B cells obtained from patients with IgAN and HCs, revealing the impact of chromatin structure alterations on aberrant gene expression in Gd-IgA1 secretion. The predicted TF, KLF4, was experimentally validated for its involvement in the expression of genes related to the intestinal immune network for IgA production. Our results suggest that IL-6 may be a major factor inducing the overproduction of nephritogenic IgA mediated by KLF4. Overall, our research revealed a chromatin-based mechanism underlying IgAN, and the TFs identified in this study merit further investigation to elucidate their importance in IgAN.

## Supplementary information

Supplementary Information

## Data Availability

Derived data supporting the findings of this study are available from the corresponding author on request. The human data are not publicly available due to ethical restrictions approved by the ethics committee of Sun Yat-sen Memorial Hospital.
