# Supplementary material for: Dynamics of chromatin accessibility governing Gd-IgA1 synthesis in B cells associated with IgA nephropathy
Source: Exp Mol Med. 2025 Jul 23;57(7):1593–606. doi: 10.1038/s12276-025-01505-1 (PMC12322228; doi:10.1038/s12276-025-01505-1)
Supplement: Supplementary file 1 — Supplementary Information [file 12276_2025_1505_MOESM1_ESM.pdf]

Supplementary Materials

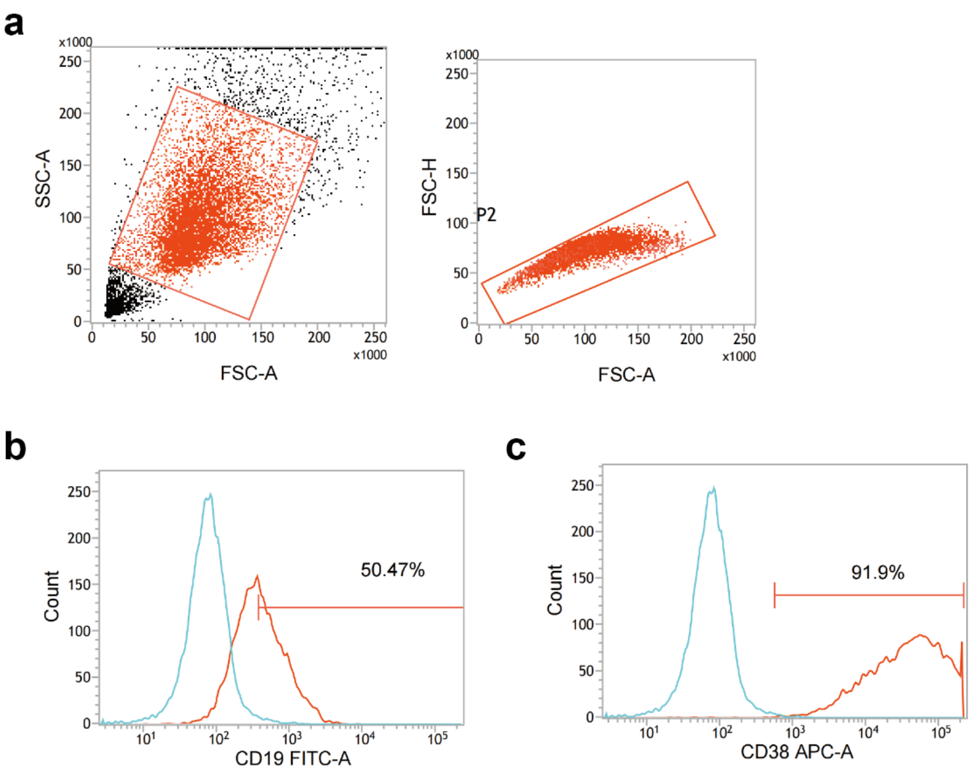

Supplementary Fig. 1 Phenotype characterization of EBV-immortalized B cells from patients with IgAN. (a) Flow cytometry was used to identify surface molecular markers of EBV-immortalized B cells from IgAN patients. (b) Representative cytometry image of CD19 expression levels on the surface of EBV-immortalized B cells from IgAN patients. (c) Representative cytometry image of CD38 expression levels on the surface of EBV-immortalized B cells from IgAN patients.

Supplementary Table 1 Primers designed for real-time PCR analysis.

Genes	Sequences
KLF4	Forward：5‘-CCCACATGAAGCGACTTCCC-3’
	Reverse：5‘-CAGGTCCAGGAGATCGTTGAA-3’
GAPDH	Forward：5‘-GGAGCGAGATCCCTCCAAAAT-3’
	Reverse：5‘-GGCTGTTGTCATACTTCTCATGG-3’
IL-6 promoter	Forward：5’-CGTGCATGACTTCAGCTTTACTC-3’
	Reverse：5’-TCATTGAGGCTAGCGCTAAGAAG-3’

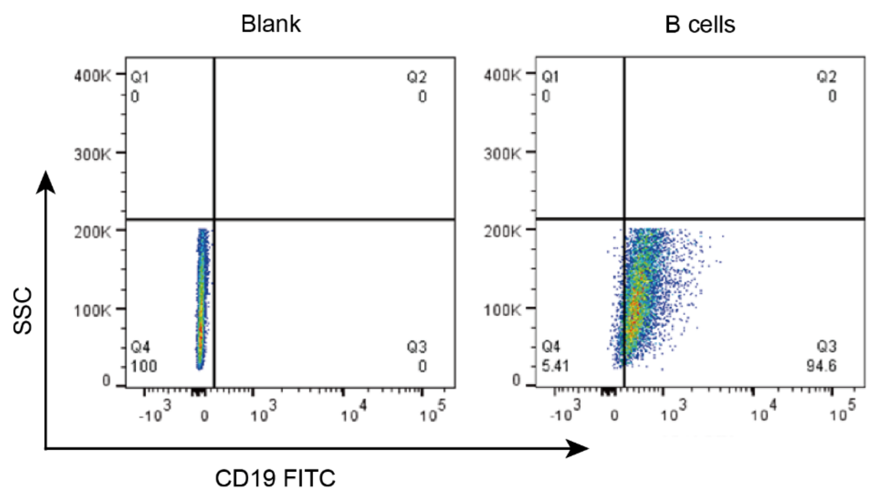

Supplementary Fig. 2 Flow cytometric identification of primary B cells from human PBMCs. The cytometry image demonstrated the CD19 expression levels of cells sorted using human CD19 magnetic beads. PBMC: peripheral blood mononuclear cells; Blank: Blank control.

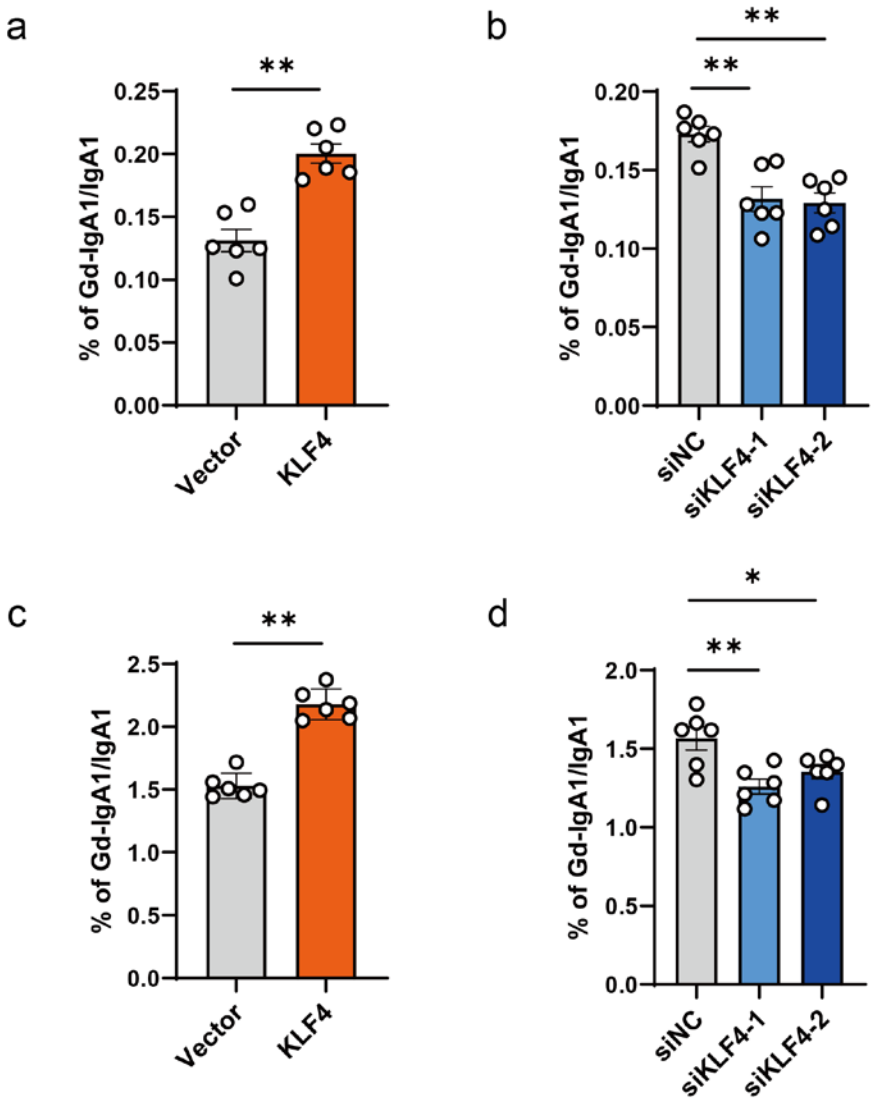

Supplementary Fig. 3 Overexpression and knockdown of KLF4 on the percentage of Gd-IgA1 in total IgA1 in *vitro*. **(a)** The percentage of Gd-IgA1 in total IgA1 was elevated in transfection of EBV-immortalized B cells with adenoviruses containing coding sequences for KLF4. **(b)** The percentage of Gd-IgA1 in total IgA1 was decreased after electroporation of synthetic siRNA targeting the human KLF4 gene or control siRNA into the EBV-immortalized B cell lines. **(c)** The percentage of Gd-IgA1 in total IgA1 was elevated in transfection of the DAKIKI cell line with adenoviruses containing coding sequences for KLF4. **(d)** The percentage of Gd-IgA1 in total IgA1 was decreased after electroporation of synthetic siRNA targeting the human KLF4 gene or control siRNA into the DAKIKI cell line. Bars represent the mean ± SEM. *$P < 0.05$, **$P < 0.01$.

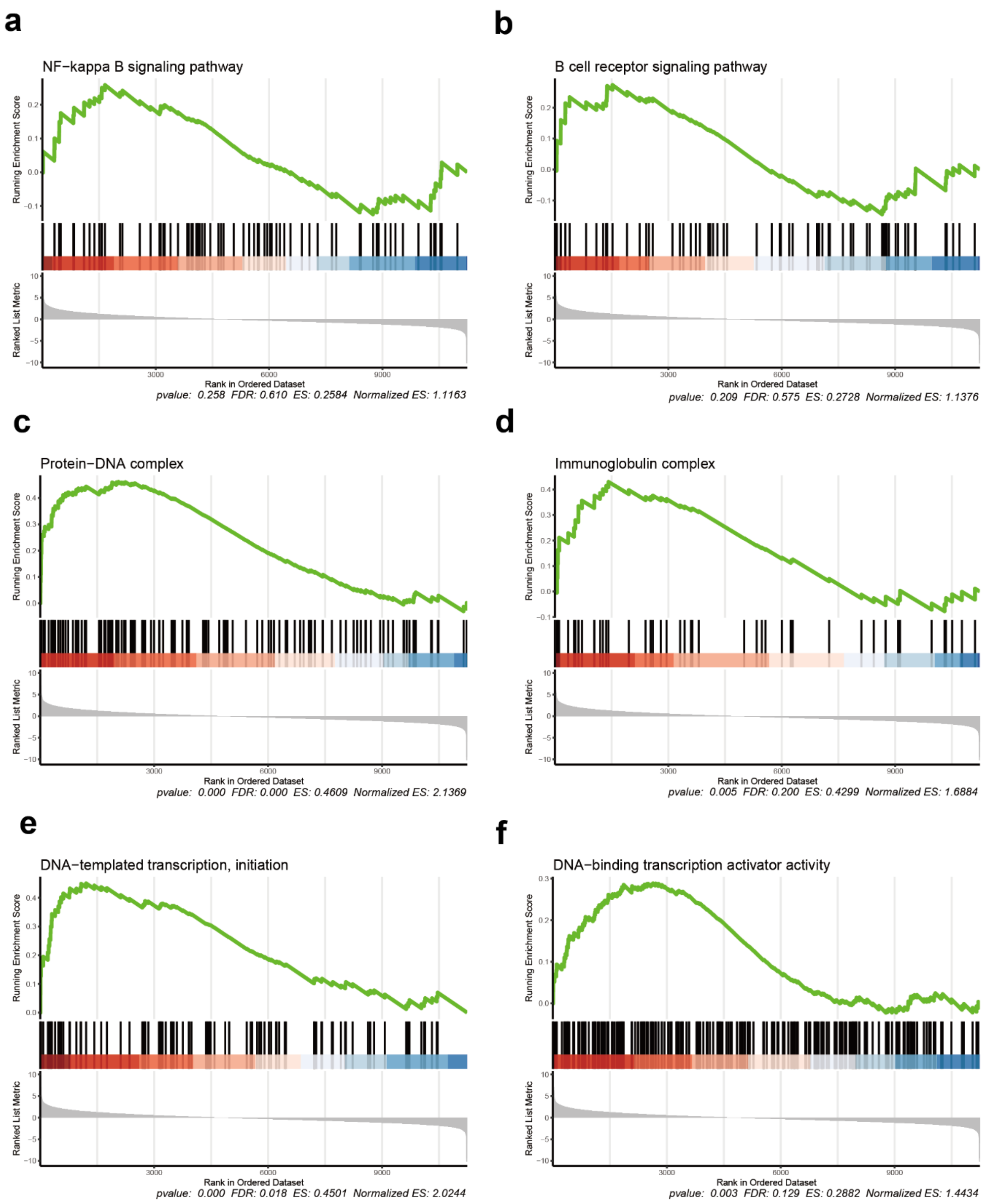

Supplementary Fig. 4 RNA-seq identified pathways enriched in KLF4-overexpressing EBV-immortalized B cells. (a) GSEA highlighting the enrichment of the NF-kappa B signalling pathway in KLF4-overexpressing EBV-immortalized B cells. (b) GSEA highlighting the B cell receptor signalling pathway enriched in KLF4-overexpressing EBV-immortalized B cells. (c) GSEA highlighting the protein-DNA complex pathway enriched in KLF4-overexpressing EBV-immortalized B cells. (d) GSEA highlighting the enrichment of the immunoglobulin complex pathway in KLF4-

overexpressing EBV-immortalized B cells. (e) GSEA highlighting the enrichment of genes related to the DNA-templated transcription pathway in KLF4-overexpressing EBV-immortalized B cells. (f) GSEA highlighting the enrichment of the DNA-binding transcription activator activity pathway in KLF4-overexpressing EBV-immortalized B cells. GSEA: gene set enrichment analysis.

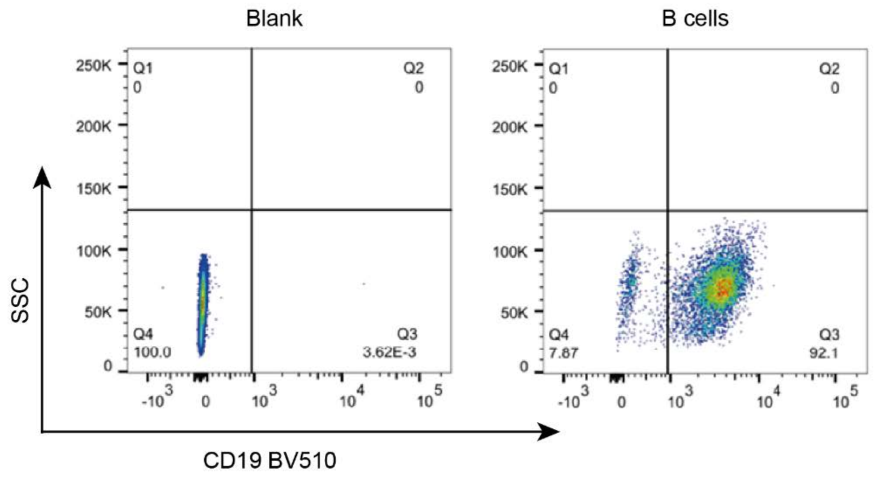

Supplementary Fig. 5 Flow cytometric identification of primary B cells from the mouse spleen. The cytometry image demonstrated the CD19 expression levels of cells sorted using mouse CD19 magnetic beads. Blank: Blank control.

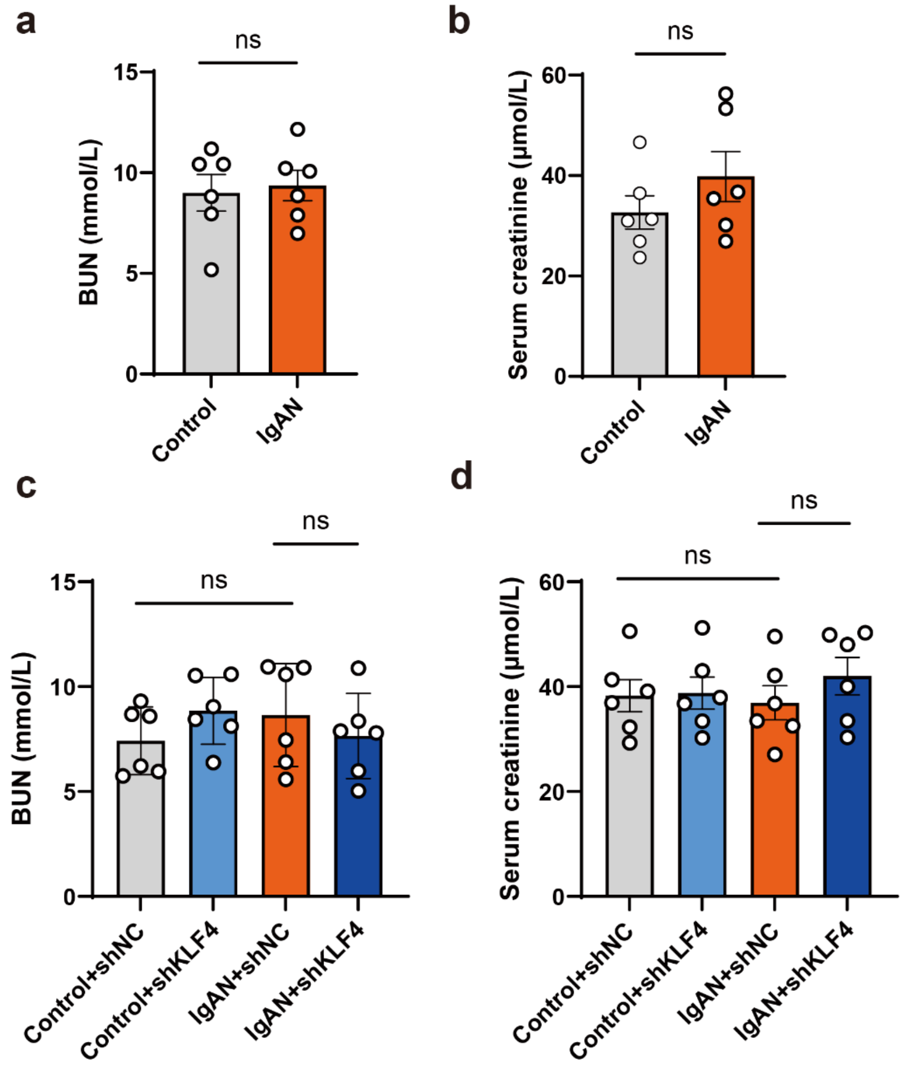

Supplementary Fig. 6 Effect of KLF4 knockdown on kidney function in IgAN mice model. (a) Serum BUN levels in the control and IgAN groups were measured. (b) Serum creatine levels in the control and IgAN groups were measured. (c) Serum BUN levels were determined for the four groups as indicated. (d) Serum creatine levels were determined for the four groups as indicated. Bars represent the mean ± SEM. **P* < 0.05, ***P* < 0.01.

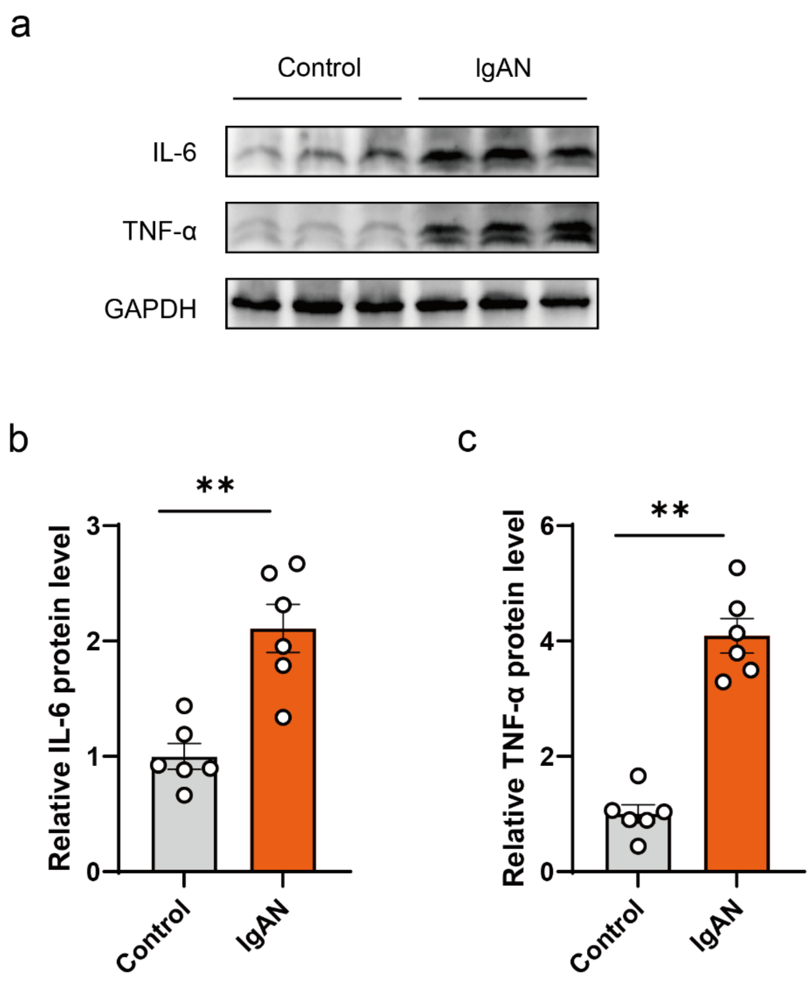

Supplementary Fig. 7 Inflammatory markers were significantly upregulated in kidney tissue from IgAN mice. (a) Western blot analysis revealed upregulation of inflammatory markers in the kidney tissue of IgAN mice. (b) Statistical results showed IL-6 protein levels in kidney tissue were also significantly higher in the IgAN group in *vivo*. (c) Statistical results showed TNF-α protein levels in kidney tissue were significantly increased in the IgAN group in *vivo*. Bars represent the mean ± SEM. *$P < 0.05$, **$P < 0.01$.
